# Home-Use and Portable Biofeedback Lowers Anxiety and Pain in Chronic Pain Subjects

**DOI:** 10.1177/15598276231221112

**Published:** 2023-12-12

**Authors:** Franklin S. Ly, Tyler Santander, Stephany Pavlov, Jiayang Zhao, Minghao Zhang, Dahyana Arroyo, Sergey Sokolovskiy, Anirudh Iyer, Yanis Yankauskas, John Chen, Michael B. Miller, Linda Petzold, Henry T. Yang, Paul K. Hansma

**Affiliations:** 1 Department of Mechanical Engineering, 8786 University of California, Santa Barbara, CA, United States; 2 Department of Psychological & Brain Sciences, 8786 University of California, Santa Barbara, CA, United States; 3 Department of Computer Science, 8786 University of California, Santa Barbara, CA, United States; 4 Department of Physics, 8786 University of California, Santa Barbara, CA, United States

**Keywords:** biofeedback, anxiety, chronic pain, portable

## Abstract

In this study, we investigated the use of novel, home-use and portable biofeedback devices in a remote program for managing chronic pain. In three separate 4-week pilot studies, participants engaged in twice-daily, 10-minute biofeedback sessions, with self-assessed reductions in anxiety and pain levels using the 6-item State-Trait Anxiety Inventory (STAI-6) and Visual Analogue Scale (VAS), respectively, in Studies 2 and 3. Among these 113 (Study 2) and 237 (Study 3) biofeedback sessions, 81 (∼72%) and 130 (∼55%) showed reductions in pain, while 93 (∼82%) and 184 (∼78%) experienced reductions in anxiety. A positive relationship was found between anxiety and pain reduction, indicating that larger reductions in anxiety correspond to larger reductions in pain. In Study 1, only anxiety reductions were measured: across 143 biofeedback sessions, 127 experienced reductions in anxiety (∼89%). Participants in all studies demonstrated reductions in baseline to final results in pain, anxiety, and showed increases in satisfaction and recovery. Our results provide strong evidence that portable biofeedback devices can enhance pain management programs by helping to alleviate anxiety and pain in individuals living with chronic conditions. This study can provide a basis for the integration of biofeedback devices into the expanding research of lifestyle and integrative medicine.

“Thermal biofeedback allows individuals to acquire knowledge regarding their capacity to modulate blood flow.”

## Introduction

Chronic pain, impacting millions of individuals globally, stands as a primary contributor to disability and diminished quality of life.^1-5^ In this paper, we investigate the effectiveness of home-use and portable biofeedback devices in helping to reduce chronic pain. Biofeedback is a mind-body technique that assists users to in gaining conscious control over a physiological process of the body, leading to improved physical and mental health.^6,7^ This approach enables the acquisition of self-regulation over physiological responses, thereby fostering relaxation and enhancing well-being.

Many studies have been conducted showing benefits to mindfulness meditation, cognitive therapy, psychophysiologic therapy, and multidisciplinary treatments for chronic pain.^8-17^ Biofeedback has been shown to be an effective non-invasive treatment option for chronic pain,^6,18-20^ and it has been demonstrated as a highly efficacious intervention for the alleviation of anxiety symptoms.^7^ By utilizing biofeedback techniques, patients may experience a reduction in pain intensity and anxiety, and improve well-being.

In a recent controlled study, 33 out of 50 participants (66%) with long-term lower back pain were randomized to receive 4 weeks of pain reprocessing therapy. At posttreatment, they were pain-free or nearly pain-free.^21^ Through pain reprocessing therapy, patients' beliefs about the causes and threat value of pain are shifted. This therapy utilized a combination of cognitive, somatic, and exposure-based techniques to assist patients in reconceptualizing their pain as a result of nondangerous brain activity, rather than peripheral tissue injury. The principles of pain reprocessing therapy have been presented in a new book, “The Way Out,” by Alan Gordon.^22^ A central component of pain reprocessing therapy involves the reduction of fear and anxiety associated with pain. Our study explores the question: what benefits can be derived from the use of home-based biofeedback devices combined with a remote group course based on this book for individuals with chronic pain?

Lifestyle medicine, a rapidly developing field of research, has emerged as a systematic approach for managing chronic diseases, with recent investigations focusing specifically on the effects of lifestyle interventions on chronic pain.^23-31^ These studies have shown efficacy towards the benefit of lifestyle medicine approach for chronic pain. Biofeedback within lifestyle medicine research has shown benefits for various health conditions.^26,32,33^ These studies serve as a basis for the field of lifestyle medicine and integrative medicine, illustrating how home-use and portable biofeedback devices can be integrated into programs. Building on this foundation, this paper presents the results of three pilot studies that investigate the effects of novel, home-use, and portable biofeedback technology in a 4-week therapy program on anxiety, pain, and satisfaction and recovery in chronic pain subjects.

## Experimental Methods

### Participants, Procedures, and Materials

All participants provided written informed consent as approved by the University of California Institutional Review Board. Demographic information is available in the Supplemental Table. The surveys used for the study can be accessed with this link: https://ucsb.co1.qualtrics.com/jfe/form/SV_3edTbDQ1pS1zmRg

#### Study 1

A group of 7 participants with chronic lower back pain or fibromyalgia for more than 6 months was recruited from referrals, posters, and emails from the Santa Barbara County Community. Inclusion in this study was based on: (1) Age above 18; (2) Proximity to UCSB for mailing purposes; (3) Access to the internet; (4) Status of experiencing chronic pain for at least 6 months. Demographic information including age, gender, and ethnicity were also collected. Once consented, participants were randomly assigned to complete the biofeedback sessions either the first two weeks of the study, or the latter two weeks of the 4-week study.

This 4-week study was entirely remote, with a 1 hour Zoom session for the entire group at the beginning and in each following week, for a total of 5 group sessions. The participants were provided with a home-use pulse and temperature biofeedback device for 2 weeks of the study and the book “The Way Out” by Alan Gordon^22^ for the entire study. The Zoom sessions focused on learning the concepts of the book with PowerPoint presentations, group sharing and discussions.

**Figure 1.**
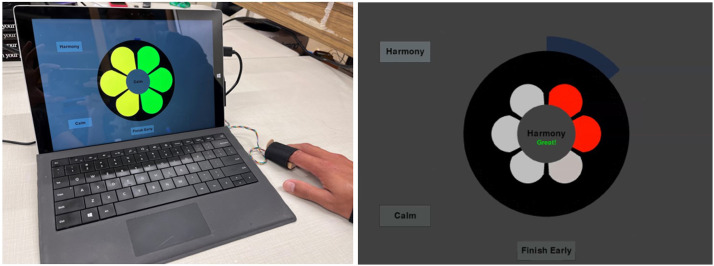
The device used in Study 1 was a home-use biofeedback device that has a temperature and pulse sensor strapped onto the finger. The data acquisition and biofeedback display is performed on a tablet. The left figure shows the display with temperature used to change the color of the segments sequentially. The right figure shows the breath pacer (segments expand and contract) with integrated magnitude of respiratory sinus arrhythmia used to change the color of the segments sequentially.

The two biofeedback methods used in this study were thermal biofeedback and heart rate variability (HRV) biofeedback. Subjects were instructed to perform 5 minutes of thermal biofeedback and then switch to 5 minutes of HRV biofeedback in each session twice a day. The device displayed the temperature with a six-segment display (Figure 1) using an absolute temperature scale. As the finger temperature increased, segments changed colors in a clockwise direction. Each color set defined a different set of temperature ranges. The heart rate variability biofeedback provided a breath pacer for subjects to follow. It used the interbeat-interval and Dynamic Phase Extraction^34^ to measure the heart rate variability, and displayed the integrated magnitude of respiratory sinus arrhythmia. The segments changed in a clockwise direction based on the value of the integrated magnitude.

The custom biofeedback device measured finger temperature with an infrared temperature sensor (MLX 90614) and pulse with photoplethysmography (PPG). The temperature sensor measures heat using infrared light without direct contact. The photoplethysmography sensor shines green light on the skin; a photodiode then captures the reflected light to detect pulse waves and measure heart rate. The visual biofeedback signal was displayed on Microsoft Surface Pro tablets (Figure 1). The microprocessor was a Wemos Lolin ESP32 board that used a serial connection to the tablets to acquire and present the biofeedback signal with a custom python software. The raw temperature and pulse data, together with pulse parameters such as the magnitude and phase of respiratory sinus arrhythmia as computed with Dynamic Phase Extraction,^34^ were sampled at 100 Hz, stored locally, and transmitted to cloud storage. When the device was connected to the internet, it transmitted the data after each session. If not, the device stored the data and transmitted the data later when the device was connected to the internet.

Anxiety levels were measured on the tablet by a six‐item short‐form of the state scale of the Spielberger State—Trait Anxiety Inventory (STAI-6),^35^ which was collected at the beginning and end of each biofeedback session.

In addition to the six‐item short‐form of the state scale of the Spielberger State—Trait Anxiety Inventory (STAI-6), before and after each biofeedback session, participants completed an online Qualtrics survey before the first group meeting and after the last group meeting. This survey contained the trait anxiety (STAI),^36^ Satisfaction and Recovery Index (SRI),^37^ McGill Pain Inventory,^38^ and questions about maximum, average and minimum pain with a Visual Analogue Scale (VAS) in the preceding week.^39^

#### Study 2

A group of 6 primarily chronic lower back pain and fibromyalgia subjects was recruited from referrals, posters, and emails from the Santa Barbara County Community. One subject did not have lower back pain but had lower pelvic pain. Inclusion in this study was based on: (1) Age above 18; (2) Access to the internet and app store; (3) Status of experiencing chronic pain for at least 6 months. Demographic information including age, gender, and ethnicity were also collected.

This 4-week study was entirely remote, with a 1 hour Zoom session for the entire group at the beginning, followed by 1 hour Zoom sessions for the entire group each following week, for a total of 5 group sessions. The participants were provided with a portable handheld temperature biofeedback device for the entire study and the book “The Way Out” by Alan Gordon.^22^ The Zoom sessions focused on learning the concepts of the book with PowerPoint presentations, group sharing and discussions. These sessions were iterated based on responses from participants in Study 1.

**Figure 2.**
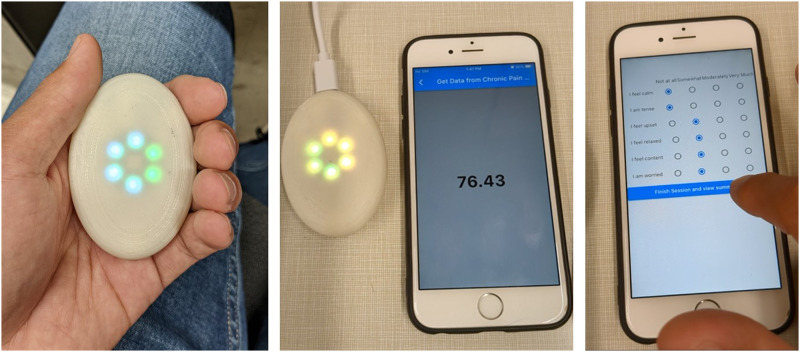
(Left) The portable biofeedback devices used in Studies 2 and 3 had a temperature sensor measuring at the palm. (Middle) The device paired with a mobile app for temperature data collection. (Right) STAI-6 and VAS were recorded before and after each session.

The biofeedback method used in this study was thermal biofeedback. Subjects were instructed to perform 10 minutes of thermal biofeedback each session twice a day. The device was portable, fit in the subject’s hand and displayed the temperature with a six-light display (Figure 2) using an absolute temperature scale. As the finger temperature increased, lights changed colors in a clockwise direction. Each color set defined a different set of temperature ranges. Data was collected through a mobile app that included measuring before and after session STAI-6 and VAS, and collected the temperature data.

In addition to the STAI-6^35^ and VAS, before and after each biofeedback session, participants completed an online Qualtrics survey before the first group meeting and after the last group meeting. This survey contained the trait anxiety (STAI), SRI, McGill Pain Inventory,^36-39^ and questions about maximum, average and minimum pain in the preceding week that were the same as Study 1.

The custom portable biofeedback device measured hand temperature with an infrared temperature sensor (MLX 90614). The visual biofeedback signal was displayed on the device with light emitting diodes (LEDs) that represented the temperature measurement. The microprocessor was a Seeed Studio nRF52840 board that used Bluetooth low-energy (BLE) to transmit data to a custom mobile app for data acquisition sampling at 50 Hz.

#### Study 3

A group of 9 participants without exclusion to type of chronic pain was recruited from referrals, posters, and emails. Inclusion in this study was based on: (1.) Age above 18; (2.) Access to the internet and app store; (3.) Status of experiencing chronic pain for at least 6 months. Location was not considered, as the devices were mailed out in this study. Demographic information including age, gender, and ethnicity were also collected.

This was a 4-week study with a 1-hour individual meeting in the week leading up to the study which was not required for all participants but was completed by the 9 subjects in the group. During this meeting which was held online or in person depending on subject comfortability, the participants were provided with and instructed on how to use the portable handheld temperature biofeedback device and how to download the app for use at home. Subjects were instructed to perform 10 minutes of thermal biofeedback each session twice a day. The rest of the study was remote with a 1 hour Zoom session offered twice a week for the following 5 weeks for a total of 5 group sessions and 1 individual session. Participants were also provided “The Way Out” by Alan Gordon which they were allowed to keep for the entirety of the study.^22^ The Zoom sessions focused on learning the concepts of the book with PowerPoint presentations, group sharing and discussions with minimal iterations made from the previous study, to include terminology encompassing different types of chronic pain, not limited to chronic lower back pain and fibromyalgia.

The biofeedback method and device were the same as in Study 2, and additionally included a breath pacer in the device. The breath pacer was visualized by the increasing and decreasing brightness of the lights and had a 3-second inhalation and 7-second exhalation period.^40^ The mobile app was the same, where the data included measuring before and after session STAI-6 and VAS, and collected the temperature data. Table 1 shows an overview of the biofeedback method and device for each study.

**Table 1.** Biofeedback Overview for Use in Studies 1, 2, and 3. The Details Include the Biofeedback Method, Duration, Device, and Surveys.

	Biofeedback Method	Biofeedback Duration	Biofeedback Device	Biofeedback Survey
Study 1	Temperature and HRV	2 weeks	Finger insert and tablet (home)	STAI-6
Study 2	Temperature	4 weeks	Handheld and phone (portable)	STAI-6 and VAS
Study 3	Temperature with breath pacer	4 weeks	Handheld and phone (portable)	STAI-6 and VAS

In addition to the STAI-6^35^ and VAS, before and after each biofeedback session, participants completed an online Qualtrics survey before the first group meeting and after the last group meeting. This survey used the trait anxiety (STAI), SRI, McGill Pain Inventory,^36-39^ and questions about maximum, average, and minimum pain in the preceding week that were the same as in Study 1 and Study 2.

### Statistical Modeling

Self-reported pain and anxiety measures were analyzed using Bayesian hierarchical models via Stan and brms in R.^41,42^ Broadly, these allowed us to assess three hypotheses: (1) that self-reported pain and anxiety decrease when comparing *pre*-biofeedback to *post*-biofeedback ratings, within each biofeedback session; (2) that the *magnitude* of the pre-post difference in anxiety correlates with the degree of change in pain, within each session; and (3) that there are longer-term changes in pain and anxiety when comparing survey measures reported at baseline to those at the end of each study period. To assess effects relevant to (1), models were specified according to the following formulae (using Wilkinson notation): y ∼ 1 + prePost + (1 + prePost | subjectID), allowing for both random intercepts and random slopes across participants (under the assumption that each participant’s pre-biofeedback ratings may be correlated with the degree of pre-post change). Similarly, for (2), models were specified according to: deltaPain ∼1 + deltaAnxiety + (1 + deltaAnxiety | subjectID). And finally, for (3), we collapsed across all three studies (because each study had a small *N* and, unlike the previous two cases estimated over repeated biofeedback sessions, each participant had only two values per survey measure) using a nested random effects structure over the model intercept to account for inter-study variability: y ∼ 1 + prePost + (1 | studyID/subjectID).

For consistency, and to avoid the possibility that any given model could be unduly influenced by arbitrary prior specifications, several key parameters for all models were estimated under the same set of weakly-informative priors:

Intercept ∼ Normal (0, 5)
Slopes ∼ Normal (0, 2.5)
Random effect *SD* ∼ Half-Cauchy (0, .5)

When correlated random effects were present, as in (1) and (2) above, we retained the default priors given by brms: Random effect *r* ∼ LKJ (1)

Lastly, we also retained the default, data-dependent priors over the residual variances, which followed: σ ∼ Half-Student-*t* (3, 0, 2.5) if the median absolute deviation (*MAD*) of the outcome measure was ≤2.5, or σ ∼ Half-Student-*t* (3, 0, *MAD*) otherwise.

Robust exploration of the posterior space for all models was performed using Hamiltonian Monte Carlo, using four independent chains with 15000 iterations each (5000 of which were used as warm-up samples). We ensured that all models properly converged to equilibrium for all parameters using classical benchmarks including: the effective sample size (considering the autocorrelation between independent posterior draws); the Monte Carlo standard error (relative to the posterior *SD*); *R*-hat (the variance ratio between each chain relative to *all* chains); and no divergent Monte Carlo transitions after warm-up. For statistical inference, we report 95% credibility intervals (i.e., using the highest posterior density interval) around the posterior median parameter estimates along with the “probability of direction,” capturing the proportion of the posterior density in the hypothesized direction, above or below zero (thus, this can be thought of as a Bayesian analogue to the frequentist *P*-value.^43^ Models (1) and (3) also include measures of effect size (analogous to Cohen’s *d*) and Bayes factors (BF10) derived using the Savage-Dickey ratio of the posterior against a point-null prior estimate of zero effect. Effect sizes were computed by taking the posterior draws and dividing them by the square root of the summed residual variances and random effects variances (as per convention for hierarchical linear models^44^; yielding a posterior distribution for each effect size. The models designed for (2) above report effect sizes using *R*^2^ for both the full model and the marginal *R*^2^ capturing variance that can be attributed to the fixed effects alone.^45^ We additionally checked for outliers using a robust criterion over the residuals: if the median absolute deviation of each individual residual was greater than three times the total *MAD* over all residuals, these data points were excluded and the model was re-fit. This resulted in 2 data points (1.77%) being removed for model (2) in Study 2 and 8 data points (3.38%) for model (2) in Study 3.

## Results

**Figure 3.**
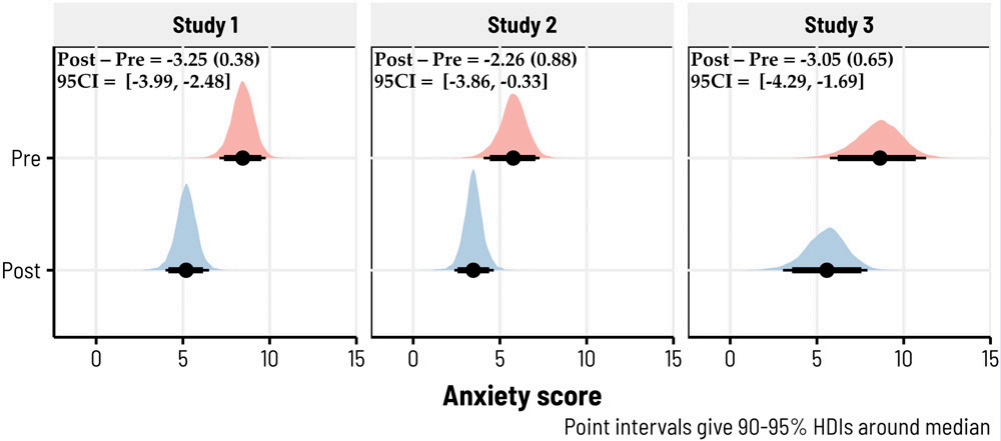
10-minute biofeedback sessions reduced anxiety across all three studies. Posterior predictive densities, derived via Bayesian hierarchical models, are shown for each study—giving the predicted estimates of pre- and post-biofeedback anxiety ratings. Anxiety was self-reported using the STAI-6, which ranged from 0 to 18. In these 493 biofeedback sessions (totaled across studies), 404 had reductions in anxiety (∼82%). The median pre/post differences, along with the posterior standard deviations and 95% credibility intervals, are given in the top left of each subplot.

We first tested the hypothesis that anxiety and pain would be reduced when comparing *pre*-biofeedback ratings to *post*-biofeedback ratings, using the Bayesian hierarchical models described under Statistical Modeling. In Study 1, we observed extremely strong evidence for a large reduction in anxiety (*d* = −1.01, 95CI = [−1.30, −.72], BF10 > 1000) under the pre-post contrast (*b* = −3.25, *SD* = .38, 95CI = [−3.99, −2.48]) with a 100% probability of being negative (i.e., such that anxiety was lower following biofeedback). Similarly, in Study 2, we observed moderate evidence for a fairly-large reduction in anxiety (*d* = −.72, 95CI = [−1.27, −.12], BF10 = 5.96; *b* = −2.26, *SD* = .88, 95CI = [−3.86, −.33]) with a 98.67% probability of being negative. Study 3 also showed extremely strong evidence for a moderate reduction in anxiety following biofeedback (*d* = −.65, 95CI = [−.99, −.31], BF10 > 100; *b* = −3.05, *SD* = .65, 95CI = [−4.29, −1.69]) with a 99.97% probability of being negative. Figure 3 highlights the posterior densities for each of these models.

**Figure 4.**
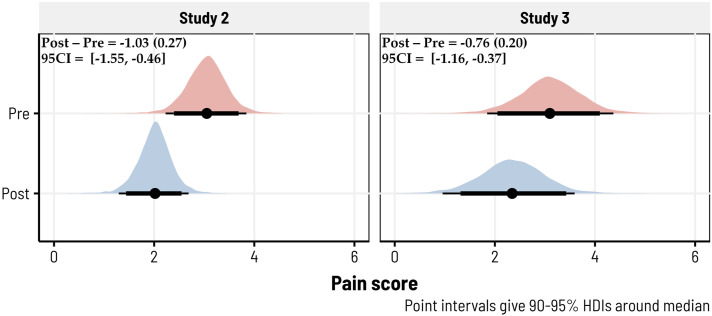
10-minute biofeedback sessions reduced pain across Studies 2 and 3. Posterior predictive densities, derived via Bayesian hierarchical models, are shown for each study—giving the predicted estimates of pre- and post-biofeedback pain ratings. Pain was self-reported using an 11-point visual analogue scale (VAS), which ranged from 0 to 10. In these 350 biofeedback sessions (totaled across studies), 211 had reductions in pain (∼60%). The median pre/post differences, along with the posterior standard deviations and 95% credibility intervals, are given in the top left of each subplot.

Changes in pain before and after biofeedback were assessed for Studies 2 and 3 (as we did not collect these ratings in Study 1). We again observed strong evidence for a moderate reduction in pain in Study 2 (*d* = −.69, 95CI = [−1.06, −.28], BF10 = 15.17; *b* = −1.03, *SD.* = .27, 95CI = [−1.55, −.46]) with a 99.79% probability of being negative. In Study 3, where we admitted participants across a wider spectrum of chronic pain types, there was a more modest reduction in pain after biofeedback, although we still observed strong evidence against the null hypothesis of no effect (*d* = −.33, 95CI = [−.54, −.14], BF10 = 24.86; *b* = −.76, *SD* = .20, 95CI = [−1.16, −.37]) and a 99.91% probability of being negative. The posterior densities for each model are shown in Figure 4.

**Figure 5.**
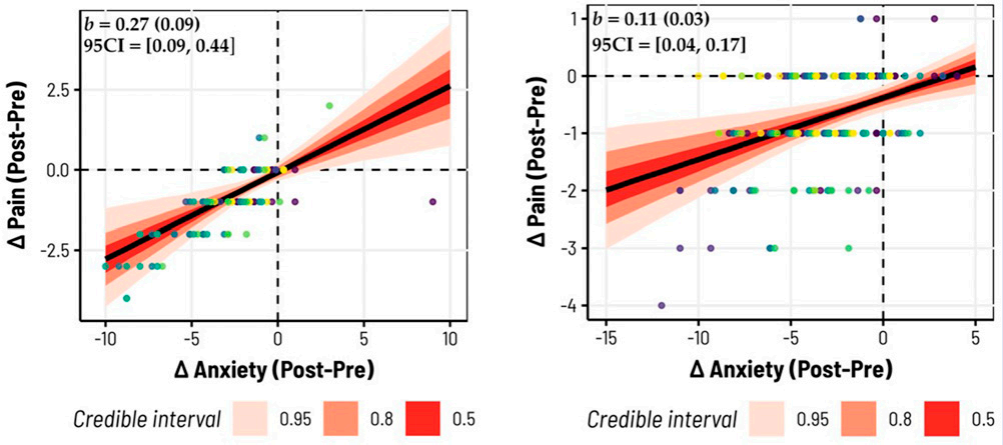
Changes in anxiety before/after biofeedback predict changes in pain. In both Study 2 (left) and Study 3 (right), the pre/post difference in self-reported anxiety following each biofeedback session corresponded with changes in pain, such that larger reductions in anxiety were accompanied by larger reductions in pain. Here we plot the raw data entered into each Bayesian hierarchical model, where different colored datapoints correspond to different subjects in each study. The population-level slopes are displayed with various uncertainty intervals (shaded in red); in the top left, we provide the posterior median estimates, their standard deviations, and 95% credibility intervals.

**Figure 6.**
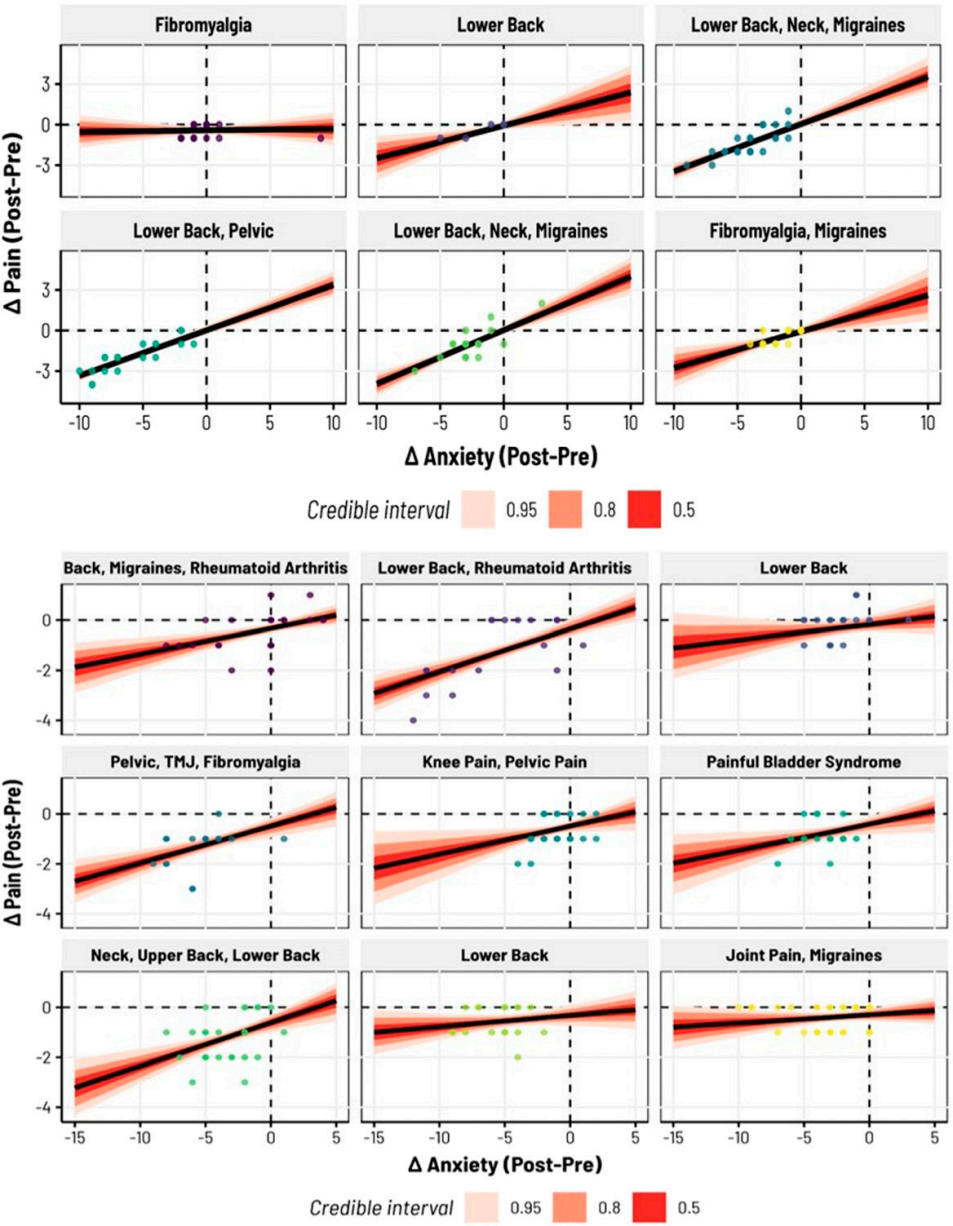
Inter-subject variability in the associations between anxiety and pain, before and after biofeedback. Here we display subject-specific estimates for the relationships between anxiety and pain reductions—that is, the random effects from the Bayesian hierarchical models shown in Figure 5. Subjects with many types of chronic pain were included in Studies 2 (top) and 3 (bottom), even subjects with Rheumatoid Arthritis, in which there was structural pain from a physical cause. For all these types of chronic pain, 10-minute biofeedback sessions usually decreased pain and anxiety (data points in the lower left quadrant).

We then sought to test the hypothesis that the magnitude of the change in anxiety (pre-/post-biofeedback) would correlate with the magnitude of the change in pain, such that larger reductions in anxiety would correspond to larger reductions in pain. This was again assessed for Studies 2 and 3. We found in Study 2 (*b* = .27, *SD* = .09, 95 CI = [.09, .44]) a 99.49% probability of the slope being positive (i.e., indicating that larger changes in anxiety were correlated with larger changes in pain). The model had substantial explanatory power considering the fixed and random effects together (*R*^2^ = .70, 95 CI = [.64, .75], adjusted *R*^2^ = .64), with the change in anxiety specifically accounting for over half of the variance (Marginal *R*^2^ = .57, 95CI = [.18, .71]). These trends are shown in Figure 5 for the overall, group-level fit, and Figure 6 highlights individual variation in slopes. In Study 3, we observed similar, albeit slightly weaker, trends (*b* = .11, *SD* = .03, 95 CI = [.04, .17]), still with a 99.63% probability of being positive. Given the greater range of pain histories represented in Study 3, this model also had weaker total explanatory power relative to Study 2 (*R*^2^ = .35, 95CI = [.26, .43]), adjusted *R*^2^ = .30) with the change in anxiety captured by the marginal *R*^2^ accounting for 15% of the total variance (95CI = [6.42 × 10^−7^, .28]). The group-level fits and individualized slopes for Study 3 are illustrated in Figures 5 and 6, respectively.

**Figure 7.**
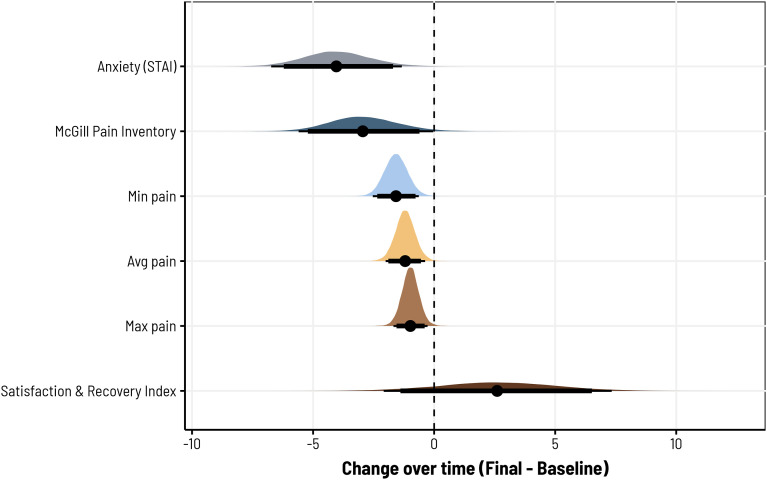
For all three studies, participants showed reductions in pain and anxiety together with increases in satisfaction and recovery from the baseline results before the study to the final results after the study. The measures obtained were maximum, average, and minimum pain using VAS, McGill Pain survey, trait anxiety from STAI, and SRI.

Finally, we examined longer-term changes in pain and anxiety by comparing several measures recorded at baseline and at the end of the study period, including STAI reports of anxiety; the McGill Pain Inventory (MPI); minimum, average, and maximum pain levels; and the Satisfaction and Recovery Index (SRI). As described previously, these measures were collapsed across studies to maximize power, while using nested random effects structures to account for variability between studies. Consistent with our pre-/post-biofeedback models, we observed strong evidence for a longer-term reduction in anxiety (*d* = −.39, 95CI = [−1.05, −.03], BF10 = 25.21; *b* = −4.01, *SD* = 1.38, 95CI = [-6.65, −1.23]) with a 99.71% probability of being negative. We also observed moderate evidence for a reduction in pain as per the MPI, although we note potential uncertainty in the estimated effect size and contrast estimate, which have credibility intervals lightly intersecting zero (*d* = −.30, 95CI = [-.61, .001], BF10 = 4.35; *b* = −2.90, *SD* = 1.42, 95CI = [−5.56, 0]). Still, for MPI, the contrast posterior suggests a 97.49% probability of being negative. With respect to participants’ minimum, average, and maximum pain ratings, models consistently revealed moderate-to-strong evidence for a reduction in each. For minimum pain, the change relative to baseline was large (*d* = −.88, 95CI = [-1.45, −.31], BF10 = 25.63; *b* = −1.57, *SD* = .49, 95CI = [−2.51, −.61]) with a 99.88% probability of being negative. For average pain, the reduction was slightly lower in magnitude (-*d* = −.74, 95CI = [-1.28, −.21], BF10 = 8.83; *b* = −1.19, *SD* = .41, 95CI = [−2.00, −.37]) with a 99.66% probability of being negative. And for maximum pain, we also observed a slightly lower reduction on average (*d* = −.52, 95CI = [−.93, −.13], BF10 = 4.51; *b* = −.98, *SD* = .36, 95CI = [−1.68, −.27]) with a 99.45% probability of being negative. However, despite these differences, we did not see evidence for a consistent increase in SRI across the three study periods, which was considerably more variable than the other measures assessed (*d* = .04, 95CI = [−.04, .15], BF10 = 1.81; *b* = 2.60, *SD* = 2.39, 95CI = [−2.12, 7.30]) and showed only an 86.16% probability of being positive. Figure 7 provides a summary of the posterior differences for each of these effects and Table 2 provides a summary of the average measures for each study.

**Table 2.** Average Baseline and Final Survey Results for Studies 1, 2, and 3. The Measures Obtained Were Maximum, Average, and Minimum Pain Using VAS, McGill Pain Survey, Trait Anxiety From STAI, and SRI.

	Study 1	Study 2	Study 3	Combined
Max pain baseline	8.0 ± 0.6	6.8 ± 2.3	7.4 ± 2.1	7.4 ± 1.7
Max pain final	7.4 ± 1.0	5.8 ± 1.0	6.2 ± 1.9	6.5 ± 1.3
Avg pain baseline	5.4 ± 2.1	4.2 ± 1.8	4.6 ± 1.3	4.7 ± 1.7
Avg pain final	3.4 ± 0.5	3.4 ± 0.8	3.4 ± 1.6	3.4 ± 1.0
Min pain baseline	3.6 ± 2.8	2 ± 1.5	3.4 ± 1.4	3.0 ± 1.9
Min pain final	.6 ± 0.8	1.0 ± 0.8	1.4 ± 1.8	1.0 ± 1.1
McGill pain baseline	22.2 ± 5.5	16.8 ± 8.7	17.2 ± 9.5	18.7 ±7.9
McGill pain final	15.4 ± 4.3	10.6 ± 9.6	14.4 ± 9.8	13.5 ± 7.9
STAI baseline	24.6 ± 4.3	25.8 ± 1.6	27 ± 5.6	25.8 ± 3.8
STAI final	15.4 ± 3.8	20.8 ± 6.9	20 ± 4.9	18.7 ± 5.2
SRI baseline	53.3 ± 15.8	59.3 ± 19.7	41.8 ± 23.6	51.5 ± 19.7
SRI final	72.5 ± 11.1	64.2 ± 9.5	64.0 ± 18.5	66.9 ± 13.0

## Discussion

Significant decreases in anxiety and pain were observed, with respective posterior medians in Studies 1, 2, and 3 being −3.25, −2.26, −3.05 for anxiety, and −1.03, −.76 in Studies 2 and 3 for pain. Additionally, a positive correlation was noted between anxiety and pain reductions, with posterior medians of .27 and .11 in Studies 2 and 3. The findings highlight the strong efficacy of biofeedback, with reductions in anxiety and pain levels observed with a probability exceeding 98.5%, and the correlation between them exceeding a 99.4% probability. Among these 113 (Study 2) and 237 (Study 3) biofeedback sessions, 81 (∼72%) and 130 (∼55%) showed reductions in pain, while 93 (∼82%) and 184 (∼78%) had reductions in anxiety. In Study 1, only anxiety reductions were measured: across 143 biofeedback sessions, 127 had reductions in anxiety (∼89%). The results provide strong evidence that portable biofeedback devices could enhance management programs by helping to alleviate anxiety and pain in individuals living with chronic pain.

Limitations of this study include the small sample sizes of participants in each study, interactions with participants conducted remotely, and the absence of a true control group in all three studies. The designs of these studies do not allow for true isolation of the factors responsible for reductions in anxiety and chronic pain. It is unclear whether the results, which compare data before and after the studies, are due to biofeedback or to the group interactions, as the effects cannot be disentangled. These pilot studies provide a foundation for further investigation, and we strongly recommend that future research include a true control group.

Thermal biofeedback allows individuals to acquire knowledge regarding their capacity to modulate blood flow. Moreover, thermal biofeedback devices have several advantages. Firstly, thermal feedback eliminates the need for costly equipment, making it a cost-effective solution. Secondly, its portable nature allows for increased convenience, enabling researchers to gather a larger amount of data per subject. Lastly, this versatility enables its utilization in a wide range of settings, further enhancing its practicality and applicability.

A question raised by this work is: How can the temporary reductions in anxiety best be used to help people overcome chronic pain and other problems? Possibilities include: 1. Reducing anxiety before, during or after somatic tracking. 2. Reducing anxiety in situations that might otherwise trigger chronic pain. 3. Reducing anxiety before, during, or after physical therapy or cognitive therapy.

As the field of lifestyle medicine and integrative medicine grows, the devices in this study can be used as a tool for research collaborations to investigate the effects of biofeedback devices in combination with other modalities for various health conditions. Utilizing the capabilities of portable biofeedback devices in remote settings, future research should investigate various methods of integrating biofeedback into programs and engage larger and more diverse samples to further validate these promising results.

## Supplemental Material


Supplemental Material - Home-Use and Portable Biofeedback Lowers Anxiety and Pain in Chronic Pain Subjects
Supplemental Material for Home-Use and Portable Biofeedback Lowers Anxiety and Pain in Chronic Pain Subjects by Franklin S. Ly, Tyler Santander, Stephany Pavlov, Jiayang Zhao, Minghao Zhang, Dahyana Arroyo, Sergio Sokolovskiy, Anirudh Iyer, Yanis Yankauskas, John Chen, Michael B. Miller, Linda Petzold, Henry T. Yang, and Paul K. Hansma in American Journal of Lifestyle Medicine.
