# Supplementary material for: Home-Use and Portable Biofeedback Lowers Anxiety and Pain in Chronic Pain Subjects
Source: Am J Lifestyle Med. 2023 Dec 12:15598276231221112. Online ahead of print. doi: 10.1177/15598276231221112 (PMC11562280; doi:10.1177/15598276231221112)
Supplement: Supplemental Material - Home-Use and Portable Biofeedback Lowers Anxiety and Pain in Chronic Pain Subjects [file sj-pdf-1-ajl-10.1177_15598276231221112.pdf]

Study #	Subject with Chronic Pain Type	Demographic Information							
		Age	Sex	Race	Duration of Pain	Maximum pain during a typical week *	Average pain during a typical week*	Minimum pain during a typical week*	# of Medications taken and type (optional)
1	Right Arm, Right Leg	30	F	Caucasian	19 years	8	5	0	0
	Fibromyalgia, Neck, Shoulders, Back, Legs, Hands	21	F	Caucasian	11 years, 4 years diagnosed	10	7	4	Unknown
	Bilateral lower back	39	M	Caucasian	8 years	9	8	7	1 methocarbamol
	Right hip	43	F	Caucasian	10 years	8	5	3	0
	Lower back, diagnosed with root compression	33	M	Caucasian	12 years	5	3	1	Unknown
	Fibromyalgia, Migraines	56	F	Caucasian	30 years	7	6	3	2 Norco, Gabapentin
	Bilateral lower back	60	M	Caucasian	40 years	8	4	2	0
2	Fibromyalgia and Migraines	20	F	Caucasian	10 years	8	5	3	Unknown
	Lower back	43	M	Caucasian	4 years	5	3	0	2 Tylenol and Ibuprofen
	Lower back, Neck, Migraines	58	F	Caucasian	Over 20 years	8	5	4	1 unspecified muscle relaxant
	Lower back, Pelvic	41	M	Caucasian	9 months	8	6	5	2: Lyrica, Gabapentin
	Lower back, Beck, Migraines	52	M	Caucasian	20 years	9	5	3	2: Botox, Aimovig
	Fibromyalgia	18	M	Asian	4 years	5	3	0	1 Motrin
3	Back, Migraines, Rheumatoid Arthritis	54	F	Caucasian	10 years, and 6 years	8	6	2	Unknown
	Lower back, Rheumatoid Arthritis	57	F	Caucasian	5 years	8	6	5	1 Ibuprofen
	Lower back	41	M	Caucasian	5 years	10	6	3	2 Tylenol, Motrin, herbal treatment
	Pelvic, TMJ, Fibromyalgia	57	F	Caucasian	Over 25 years	10	7	6	1 Celebrex, herbal treatment
	Arthritis, Pelvic Pain	60	F	Caucasian	Over 5 years	3	3	3	Unknown
	Painful Bladder Syndrome	28	F	Caucasian	1 year	8	6	2	3: Uribel, Ibuprofen, Naproxen
	Neck, upper, and lower back	23	F	Asian	3 years	7	4	2	0
	Lower back	45	F	Black	2 years	6	4	3	0
	Joint Pain, Migraines	48	F	Caucasian	Over 20 years	10	8	7	Unknown

Supplemental Table 1. Demographic information for studies 1, 2, and 3.

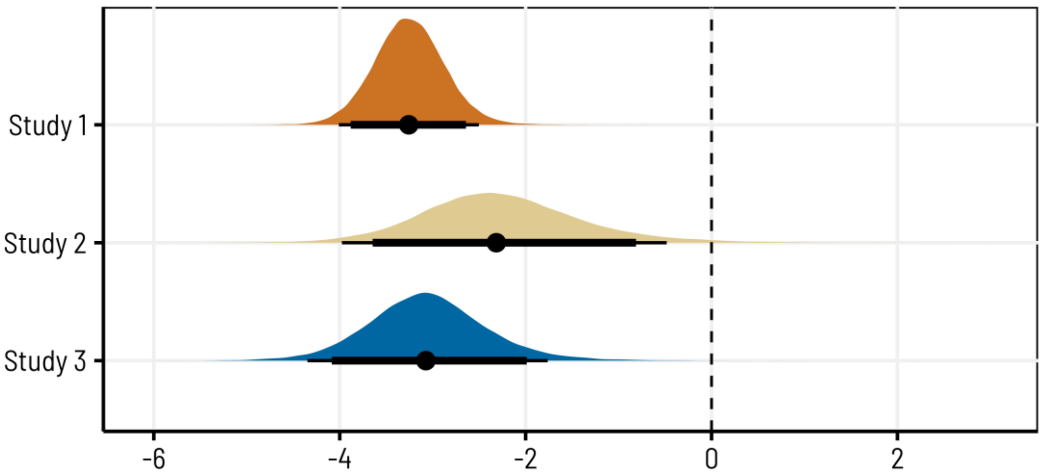

Average effect of biofeedback on anxiety (Post-Pre)

Point intervals give 90-95% HDIs around median

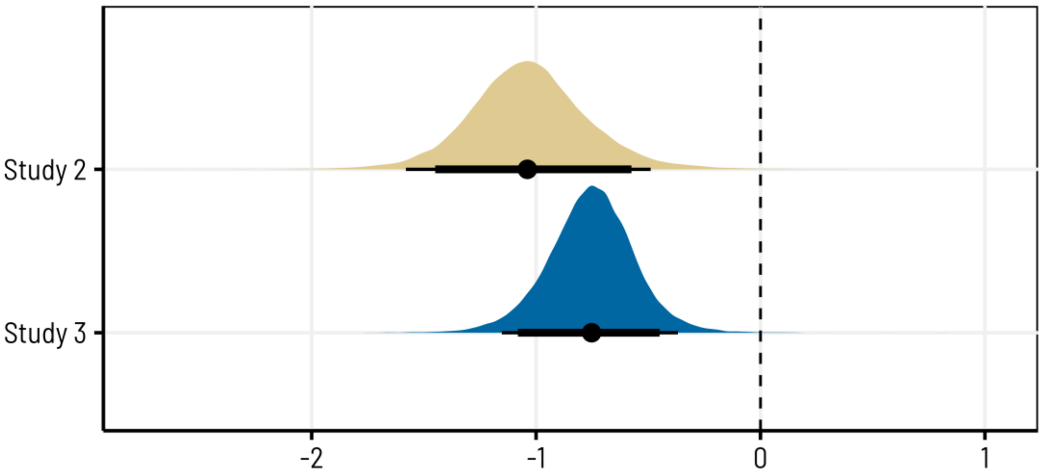

Average effect of biofeedback on pain (Post-Pre)

Point intervals give 90-95% HDIs around median

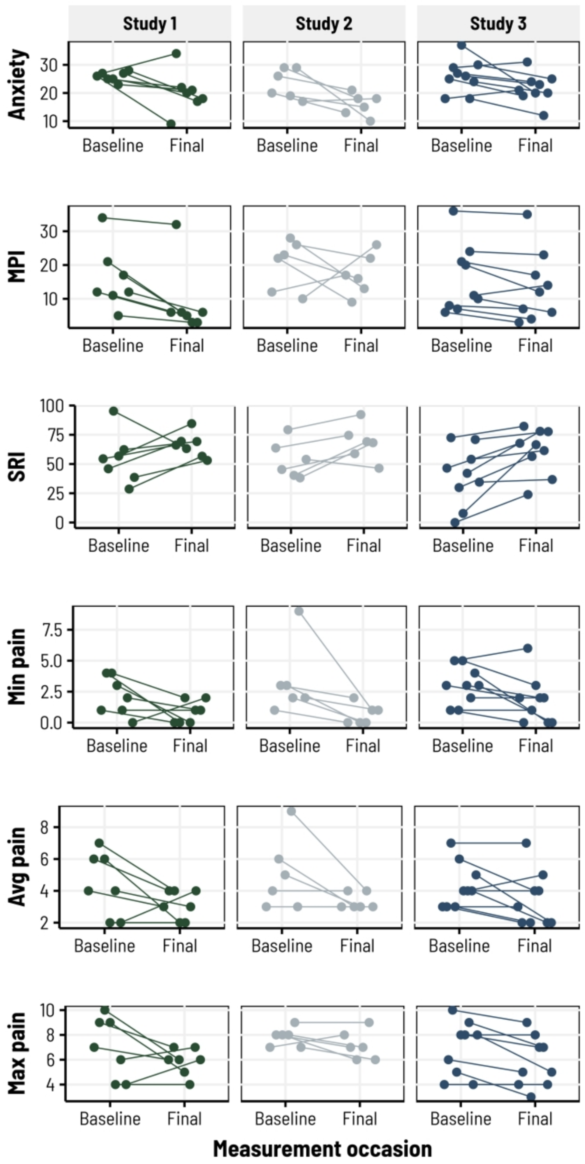

Study 1
Study 2
Study 3
Anxiety
MPI
SRI
Min pain
Avg pain
Max pain
Baseline
Final
Measurement occasion
